# Recent Treatment of Aural Diseases

**Published:** 1872-05

**Authors:** 


### RECENT TREATMENT OF AURAL DISEASES.

Dr. Charles E. Rider read before the City Medical Society of Rochester an essay on the above subject, an abstract of which is published in the Buffalo *Medical and Surgical Journal*, October, 1871:

We may for the sake of convenience study the disorders of hearing as they affect the external, middle, or internal ear.

1st. Diseases of the external ear—Eczematous Inflammation. We meet this affection quite frequently, especially among badly nourished children, often existing just back of the auricle.

Locally, I applied the following ointment:

R.—Yellow oxide of mercury, . . . gr.xx.
Simple cerate, . . . . . . oz.j.—M.

Internally, I gave good nourishment and tonics. Where the eczema is rather dry, the best remedy perhaps is Fowler's solution of arsenic; but where the eczema is more moist, I have used the following formula with much benefit:

R.—Nitric acid, . . . . . . dr.j.

Epsom Salts, . . . . . . oz.j.

Water, . . . . . . , O j.—M.

Give a tablespoonful three times a day.

Otitis Externia (diffuse otitis), commonly shows itself by an exfoliation of cuticle, with a slight purulent discharge. Sometimes, however, there may exist merely redness and dryness of the membrane, without any discharge; or even the disease may show an absence only of the normal amount of cerumen. For this trouble we must correct any constitutional vice. Astringents are indicated for local use.

Furuncular Inflammation.—This affection is extremely painful and persistent, and often very difficult to cure. As soon as one begins to push up toward the centre of the meatus, immediately puncture it with a sharp, narrow bistoury. This generally relieves the pain; hot fomentations may follow. Dr. Von Troeltsch recommends the use of arsenic in this disease.

Dr. W. W. Ely has failed to obtain benefit in these cases by freely administering quinine and producing cinchonism; but where the severity and number of these furuncles are excessive, he has seen the following treatment invariably break up their course:

R.—Hydrargyri chlorodi corrosiv, . . . gr.j.
Fl. ext. sennæ et jalapæ, . . . oz.j.—M.

Sig.—dr.j. ter die.

Accumulation of Inspissated Cerumen.—Against this we have no prophylactic, and if removed, it will usually reaccumulate at intervals of a year or more. With a syringe, as, for instance, Mattison's, *gently* throw into the meatus a continuous stream of water. This will generally soften and wash out the mass. Always, ere any treatment is used, be sure that there is no abnormal amount of wax present, as injections of even warm water in a healthy ear may cause injury. Never use any instrument to remove the cerumen, as the membrana tympani is very easily injured.

Foreign Bodies in the external Meatus.—Always first examine carefully, to ascertain if such foreign body is in the meatus. Then remove with the syringe and warm water if possible, if not, use any suitable instrument, but only under the eye. Never introduce any instrument unless the eye sees its every motion.

Ear Ache.—Make warm water applications, or hot chammomile fomentations.

2d. The membrana tympani is seldom diseased alone, but it is often involved in inflammations occurring in the external or middle ear.

3d. Diseases of the Middle Ear.—The cavity of the middle ear is lined by mucous membrane, which is a continuation through the eustachian tubes of that of the pharynx. Inflammation of this membrane is by far the most common disease of the ear.

Acute Otitis Media.—Almost every one, at some time, suffers from one or more slight attacks of otitis media. It is in fact a "cold" in the ear. We have slight pain, redness of membrana tympani, slight deafness, sensation as of water in the ear. Therapeutically, we treat it as we would a common cold; keep quiet, and apply to neck and feet derivative remedies.

Chronic Otitis Media.—This comes on very slowly, increasing very gradually; may result from acute repeated attacks of acute inflammation, but more often is slow and chronic from its inception. In this complaint quite frequently the patient does not notice any trouble until the membrana by repairing is much thickened, and the hearing considerably impaired. If the hearing progressively grows worse, or if it has no remissions or periodical seasons of comparative improvement, we have but little hope from any treatment, as any and all therapeutical measures seem to fail in improving hearing.

Subacute Inflammation.—We may, however, speak of what we might call subacute otitis media. In this form we have repeated attacks of acute inflammation, each leaving the ear worse than they found it, and although perhaps to a skilled observer the membrana typani may appear somewhat less thickened than in the last described disease, still the diagnostic distinction is found in the fact that the hearing varies, being sensibly better at some times than at others, and in this variety treatment is often very benefical, perhaps even curative. Here, if we find co-existing naso-pharyngeal catarrh, the use of the atomizer is indicated. Probably the best remedy to use therewith is a saturated solution of chlorate potassa. Also we frequently are obliged to use the Eustachian catheter. (I much prefer the hard rubber catheter, to which, by heat, any desired curve can be given.) Its use is not a painful, but it is often an exceedingly delicate operation, as we so frequently meet with septa nasi, which are so bent that it is very difficult, almost impossible, to introduce it successfully. Also in conjunction with the catheter, or politzer apparatus, we often find advantage in throwing up medicated vapors, as that of tincture of iodine or tincture of iron ; and even in blowing up finely powdered alum or iodoform. I consider it, however, more than dangerous to try to throw up solutions into the middle ear.

Purulent Otitis media.—Often a sequelar of exanthematous diseases, and frequently a result of other irritating causes, in those inheriting bad constitutions. The discharge is oftentimes very offensive, and the disease is always a dangerous one. Often it causes necrosis of bone, and may go on to form fistulous openings through the mastoid bone, or even into the cranial cavity; and hearing is always more or less impaired, and that permanently.

Treatment.—While we correct any constitutional vice, and also as a rule administer tonics, our therapeutical reliance is mainly placed on the local use of some of the astringents ; alum is the best; then come sulphate of zinc, sulphate of copper, and nitrate of silver. When in the course of this disease we have deep seated, persistent pain, we may infer that the mastoid cells are invaded, and be called on to trephine the mastoid process. In purulent disease of the middle ear I must strongly insist on the necessity that the physician should apply the local treatment personally, because no amount of instruction will enable the patient himself, or his friends, thoroughly to cleanse out the ear and apply the remedy. Also, after perfectly cleansing the ear by warm water, he must keep the Eustachian tube open ; thus he can blow through the medicated solution after its introduction into the ear, and thus secure its application to every point of diseased membrane, on which contact the efficacy of the treatment *alone* depends. Occasionally also, in this trouble, after the discharge is stopped, we find that artificial ear drums decidedly improve the hearing. If we find quite a large aperature in the ear drum, we must not despair; as in children, and especially in cases of recent date, even large sized apertures are frequently filled up by a cicatrical tissue; and the hearing eventually becomes greatly improved.—*Half-Yearly Compendium.*

